# In Vitro Evaluation of Novel Nano-Sized Colloidal Assemblies Incorporating Hydrophobic Tobramycin Ion Pairs: Enhanced Cellular Uptake with Preserved Antimicrobial Activity Toward Oral Drug Delivery

**DOI:** 10.3390/molecules31122139

**Published:** 2026-06-17

**Authors:** Khaled Husam Khaled, Ahmad Saleh Malkawi, Azhar Saleh Malkawi, Razan Haddad, Nasr Alrabadi, Rana Abu-Dahab, Amal Ghaleb Al-Bakri, Airemwen Collins Ovenseri

**Affiliations:** 1 Faculty of Pharmacy, Cyprus International University, Nicosia 99258, Cyprus; acollins@ciu.edu.tr; 2 Department of Pharmaceutical Sciences, Faculty of Pharmacy, Jadara University, Irbid 21110, Jordan; razan.haddad@jadara.edu.jo; 3 Department of Pharmaceutical Technology, Faculty of Pharmacy, Jordan University of Science and Technology, Irbid 22110, Jordan; azharmalkawi5@gmail.com; 4 Department of Pharmacology, Faculty of Medicine, Jordan University of Science and Technology, Irbid 22110, Jordan; nnalrabadi@just.edu.jo; 5 Department of Biopharmacy and Clinical Pharmacy, The University of Jordan, Amman 11942, Jordan; abudahab@ju.edu.jo; 6 Department of Pharmaceutics and Pharmaceutical Technology, School of Pharmacy, The University of Jordan, Amman 11942, Jordan; agbakri@ju.edu.jo

**Keywords:** nanocarriers, nanoemulsions, tobramycin, cellular uptake, drug delivery, antimicrobial activities

## Abstract

Tobramycin is a highly hydrophilic aminoglycoside antibiotic with limited cellular permeability and negligible oral bioavailability, necessitating parenteral administration. This study aimed to develop drug delivery systems based on nano-sized colloidal assemblies (NCAs) incorporating tobramycin ion pairs to enhance its lipophilicity, potential for transition to the oral route, and antimicrobial activities. Tobramycin was ionically paired with oleic acid, lauric acid, and fluorescein and formulated into NCA preconcentrates (F1–F5) using combinations of Tween 80, DMSO, and propylene glycol. The resulting formulations formed stable nanodroplets upon dilution (9.50–16.30 nm) with narrow size distributions (polydispersity index; PDI < 0.3) and moderate negative zeta potentials (−4.99 to −11.13 mV). In vitro release studies indicated sustained drug release for ion-paired systems compared to the rapid release of free tobramycin. Cytotoxicity evaluation in Caco-2 cells demonstrated high biocompatibility at 1:10,000 and 2:10,000 dilutions, while concentration-dependent toxicity at higher doses suggested enhanced intracellular delivery. Cellular uptake studies revealed significantly higher tobramycin internalization (*p* < 0.001) from formulations F1–F3, with uptake values in the range of 81.76–96.14% compared to free drug, which showed zero or negligible uptake. Fluorescein-labeled formulations (F4 and F5) further confirmed enhanced uptake, demonstrating strong intracellular fluorescence. This was supported by visual observation, UV–Vis absorbance (70.5–84.8% relative to positive control), and confocal microscopy imaging. Antimicrobial activities against *P. aeruginosa* and *S. aureus* were comparable between formulations F1–F5 and free tobramycin (inhibition zones of 16–18 mm), utilizing the same tobramycin concentration in the diluting medium. These findings validate the effectiveness of the formulated NCAs in facilitating intracellular delivery of tobramycin while preserving biocompatibility and similar antimicrobial activities. Moreover, the uptake of fluorescein provides indirect evidence supporting the enhanced internalization of tobramycin in analogous ion-paired formulations. This strategy holds promise for overcoming intestinal barriers and improving oral bioavailability, potentially enabling the transition of tobramycin from parenteral to oral administration.

## 1. Introduction

Oral drug delivery is the preferred administration method due to its non-invasive nature, convenience, cost-effectiveness, and typically enhanced patient adherence relative to parenteral therapy [1]. Successful oral absorption relies on an optimal equilibrium among drug solubility, gastrointestinal stability, and intestinal permeability; numerous therapeutically significant compounds do not attain adequate bioavailability following oral administration due to permeability-related barriers [1,2]. The intestinal mucosal barrier poses a significant difficulty since the mucus gel layer can impede the diffusion of therapeutic molecules and nano-sized drug delivery systems due to steric, electrostatic, hydrophobic, and hydrogen-bonding interactions [2,3]. Moreover, epithelial tight junctions restrict paracellular transport, while efflux transporters such as P-glycoprotein may diminish intracellular drug accumulation by translocating ingested molecules back to the gut lumen [4,5]. Consequently, many formulation strategies have been explored to surmount intestinal barriers, including mucus-interacting nanodroplets, permeation enhancers, lipid-based delivery systems, and nano-sized colloidal carriers aimed at enhancing epithelial contact and drug transport [2,3,4,5].

Nano-sized drug delivery formulations gained major interest among nanoscale delivery systems due to their diminutive droplet size, elevated interfacial surface area, and capacity to enhance drug dispersion and interaction with the intestinal mucosa [6,7]. Nano-sized drug delivery systems are stable dispersions, either thermodynamically or kinetically, consisting of oil and aqueous phases, which are stabilized by surfactants and co-surfactants. The nanoscale dimensions of these systems may promote interaction with the mucosal surface and optimize medication distribution at the absorption interface, thereby improving the permeability performance of weakly permeable compounds [2,6,7].

The Biopharmaceutics Classification System (BCS) categorizes drug substances into four classes based on their aqueous solubility and intestinal permeability: Class I (high solubility/high permeability), Class II (low solubility/high permeability), Class III (high solubility/low permeability), and Class IV (low solubility/low permeability). This paradigm is extensively employed to forecast oral absorption constraints and to inform formulation design for inadequately absorbed pharmaceuticals. Aminoglycosides are a significant class of cationic antibiotics exhibiting strong efficacy against susceptible bacterial pathogens, especially Gram-negative bacteria; however, their high polarity and numerous ionizable amino groups typically limit passive membrane diffusion following oral administration. Thus, the oral administration of aminoglycosides presents a significant formulation difficulty. Tobramycin is a significant aminoglycoside antibiotic utilized for treating Gram-negative infections; nevertheless, it is rarely offered in a traditional oral systemic formulation and is predominantly delivered through parenteral, inhalational, or topical methods [8]. Tobramycin is typically regarded as a prototypical BCS Class III chemical, distinguished by its high-water solubility and low membrane permeability [8]. Tobramycin is very soluble in water, with an aqueous solubility of around 53.7 mg/mL; yet, it demonstrates very low lipophilicity, with predicted log *p* values of −5.8, indicative of its highly polar polycationic structure. This physicochemical characteristic significantly restricts passive diffusion via intestinal lipid membranes following oral treatment [8,9,10]. In highly hydrophilic substances, reduced membrane permeability may further limit intracellular drug accumulation, hence decreasing apparent oral absorption [11].

Hydrophobic ion pairing (HIP) is an effective approach for altering the apparent physicochemical characteristics of highly polar medicines without covalent alteration [12,13]. This method involves an ionic medication creating a reversible electrostatic combination with a counterion of opposite charge, leading to diminished polarity and enhanced compatibility with lipid-based delivery systems [12]. Prior research has shown that HIP can improve drug integration into lipidic nano-sized drug delivery systems and influence the release dynamics of hydrophilic substances [12,13,14]. Consequently, HIP can augment the lipid solubility of tobramycin and extend its retention in nano-sized drug delivery systems intended for oral administration [15].

Our previous research has shown that nanosized surfactant-based oral delivery systems can be designed to optimize mucus dispersion and increase cellular uptake by optimizing surface characteristics and formulation composition [16]. Subsequent research has shown that self-emulsifying nanosystems can augment intestinal mucus penetration and enhance medication delivery efficacy [17]. Furthermore, we have recently reported optimized mixed micellar systems consisting of surfactants and co-solvent combinations that maintained nanoscale dimensions and enhanced in vitro delivery efficacy [18]. This study utilized a technique based on formulating nano-sized colloidal assemblies (NCAs) to include tobramycin as a hydrophobic ion pair within a lipophilic surfactant matrix, employing Tween 80 and co-solvent components to improve solubility, dispersion, and stabilization in aqueous environments.

This work presents a proof-of-concept evaluation of NCA’s capacity to improve the cellular permeability performance of a highly polar, weakly penetrating medicinal molecule. Tobramycin was used as a model chemical to investigate barrier incompatibility and assess whether hydrophobic ion pairing, in conjunction with NCA design, could enhance compatibility with lipidic domains while maintaining antimicrobial efficacy. This study aimed to develop and characterize NCAs incorporating hydrophobic ion pairs of tobramycin, with a comprehensive evaluation of their physicochemical properties, in vitro release, cytocompatibility, cellular uptake, and antimicrobial activity, in order to support the development of an efficient oral drug delivery platform and provide a foundation for future in vivo translation of these in vitro-validated nanosystems for a highly polar antibiotic.

## 2. Results and Discussion

### 2.1. Characterization of the Developed NCAs

**Figure 1:**
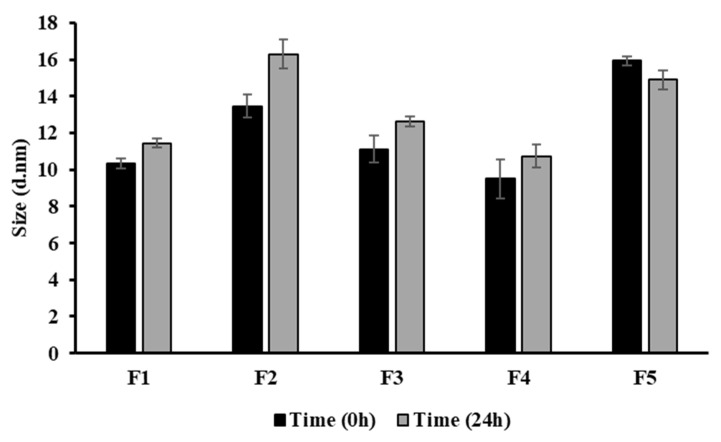
The hydrodynamic particle size of the formulations F1–F5 was determined right after they were diluted (0 h) and again after being stored at 37 °C for 24 h.

**Figure 2:**
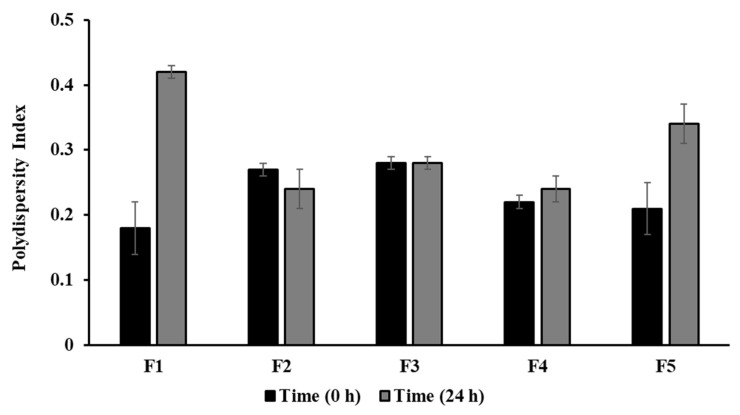
The PDI values of the formulations F1–F5 were determined right after they were diluted (0 h) and again after being stored at 37 °C for 24 h.

Previous research has widely employed dynamic light scattering (DLS) characterization to confirm the formation of nano-sized colloidal delivery systems by determining particle size and size distribution [8,13]. The hydrodynamic particle size (Figure 1) of the prepared formulations (F1–F5) was determined using 1:100 dilutions in a physiological buffer (pH 7.4) at 37 °C right after the preparation (0 h) and the storage time (24 h). The mean hydrodynamic diameter was found to be in the nanoscale range of 9.50 ± 1.05 nm to 16.30 ± 0.79 nm in all formulations. The results are a sign of the creation of nano-dispersed systems. The hydrophobic ion-pair formulation based on oleic acid (F1) and the hydrophobic ion-pair formulation based on lauric acid (F2) had a mean particle size of 10.34 ± 0.25 nm and 13.46 ± 0.64 nm at 0 h, respectively. The F3 non-ion-paired formulation had an intermediate size of 11.12 ± 0.75 nm. Among the formulations containing the ion pair of tobramycin with fluorescein, F4, with a 9.50 ± 1.05 nm mean droplet diameter, showed the smallest size, whereas F5 resulted in a 15.93 ± 0.23 nm mean droplet size. Following 24 h of incubation and shaking, only small changes in particle size were noted in all formulations, which demonstrates that there was reasonable colloidal stability under the tested conditions. The PDI values indicate that all formulations exhibited narrow size distributions at 0 h (PDI < 0.3), reflecting well-defined and homogeneous systems. Over the measurement duration, F2, F3, and F4 maintained PDI values below 0.3, confirming consistent dispersion stability (Figure 2). Although F1 and F5 showed slight increases in PDI at 24 h, their distributions remained relatively uniform with no evident disruption of peak profiles, suggesting only minor broadening rather than instability. Importantly, all PDI values remained within acceptable limits reported in the literature (PDI < 0.5), supporting the overall stability of the formulations [19]. Particle size is a critical design parameter in oral nano-sized drug delivery systems, as it governs interactions with gastrointestinal mucus and the intestinal epithelium, thereby directly influencing mucosal penetration and cellular uptake. Reduced particle size enhances diffusivity through the mucus layer and promotes closer contact with epithelial surfaces, facilitating improved internalization. Previous studies have demonstrated that nanosize, together with surface characteristics, dictates mucus adhesion, intestinal residence time, and transport behavior. In this context, the relatively small and stable particle size achieved in the present formulations is expected to support efficient mucosal permeation while preserving colloidal integrity during short-term storage. Additionally, the inclusion of Tween-based surfactants within the nanosystems may further enhance epithelial uptake, as will be discussed in a later section [2,3,4,20].

**Figure 3:**
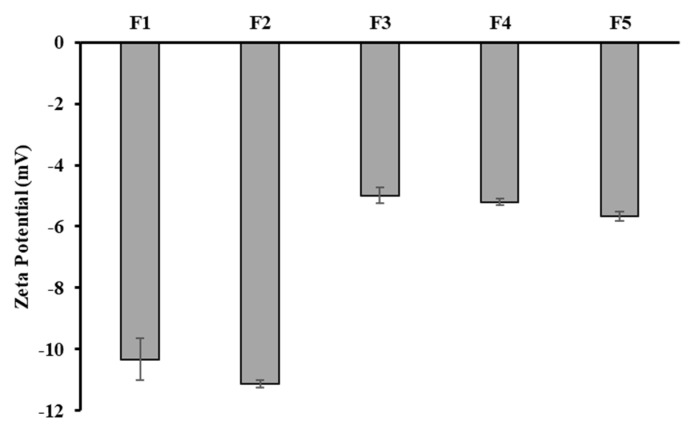
The measured zeta potential values of the tested formulations F1–F5 at 37 °C, subsequent to dilution in a physiological medium at pH 7.4.

The zeta potential of all of the formulations (F1–F5) was between −4.99 ± 0.25 mV and −11.13 ± 0.12 mV, meaning that each system had a moderate negative surface charge (Figure 3). Comparatively, the formulations F1 and F2 had more negative zeta potential values as compared to the non-ion-paired formulations. This indicates that the interfacial properties of the dispersed systems were changed by the presence of fatty acid and fluorescein counterions, masking the cationic properties of tobramycin’s primary amino groups. Zeta potential is a widely utilized parameter for assessing surface charge and predicting the colloidal stability of nanoscale dispersions. In systems stabilized with nonionic surfactants such as Tween 80, absolute zeta potential values of approximately ±30 mV or higher are generally associated with strong electrostatic repulsion, whereas lower values may still confer adequate physical stability due to steric stabilization mechanisms [21,22]. In the present study, the comparatively lower zeta potential values observed for F1 and F2 relative to the non-HIP systems are consistent with modifications in surface and interfacial characteristics following hydrophobic ion pairing (HIP). HIP is a reversible, non-covalent interaction between ionizable hydrophilic drugs and oppositely charged counterions in aqueous media, leading to increased apparent lipophilicity and transient modulation of formulation behavior [23,24]. This interaction can contribute to short-term colloidal stabilization, primarily through steric effects. The developed systems exhibited preserved nanometric size distributions, stable PDI values over 24 h, and moderate negative zeta potentials following aqueous dilution, supporting the formation of physically stable colloidal nanostructures. Although the systems may share characteristics with mixed micellar or SMEDDS-like assemblies due to their composition and small particle size, the maintained nanoscale dimensions and colloidal stability support their suitability as nano-based delivery systems [25,26]. Despite the relatively lowered zeta potential values depicted for the developed NCAs, they showed a sufficient colloidal stability verified over a duration of 24 h of size measurement. It is believed that steric stabilization contributed by the presence of the nonionic surfactant Tween 80 rather than the zeta potential-attributed electrostatic repulsion represented the dominant colloidal stabilization mechanism. In this effect, Tween 80 generates a protective hydrophilic interfacial layer around the colloidal structures and reduces aggregation despite limited electrostatic repulsion. The contribution of co-solvents and the spontaneous self-assembled nature of the systems may also support colloidal stabilization [18,27,28]. Overall, these findings suggest that interfacial properties in the presence of a stabilizing surfactant can be effectively tuned via HIP while preserving acceptable short-term stability of the nanosystems.

### 2.2. Fourier Transform Infrared (FTIR) Analysis

**Figure 4:**
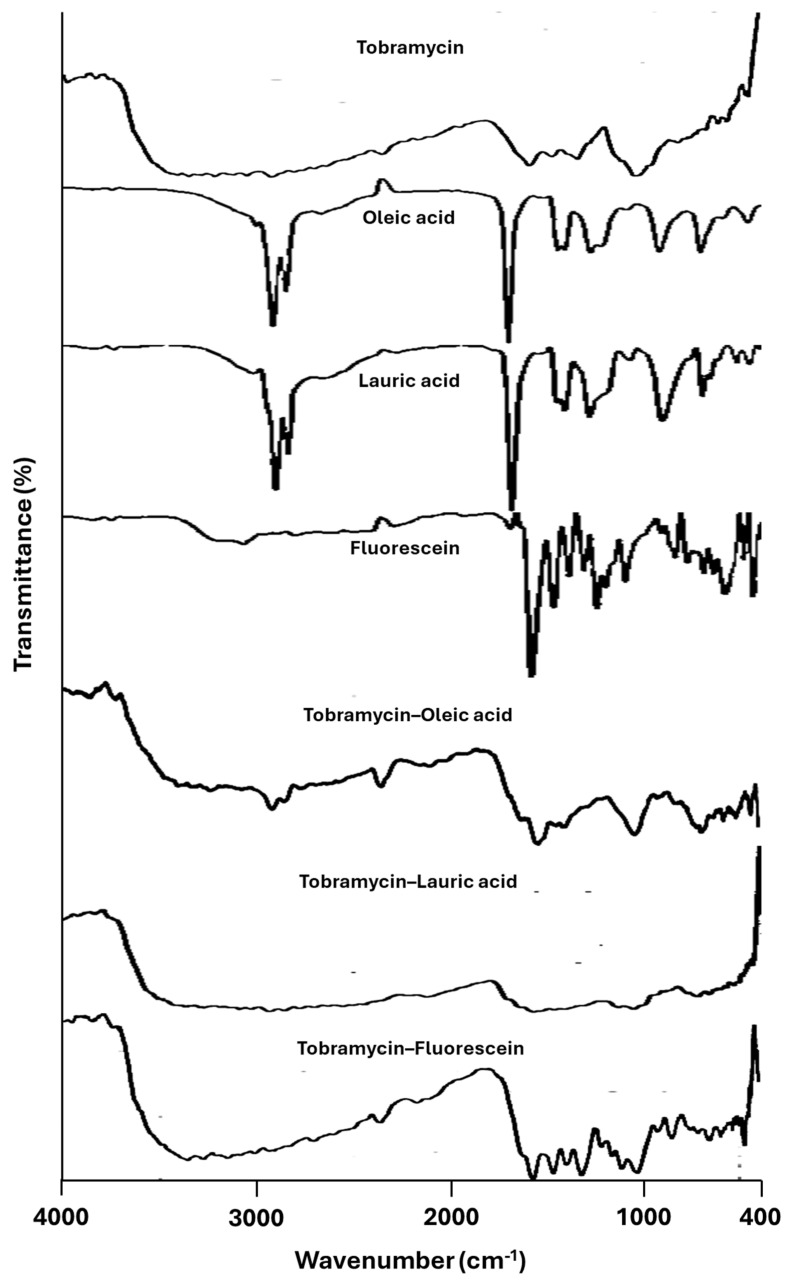
FTIR spectra of tobramycin, oleic acid, lauric acid, fluorescein, and the target ion pairs.

The formation of hydrophobic ion pairs was qualitatively evidenced by immediate visible interactions upon mixing the charge-equimolar counterparts in DMSO. This included substantial precipitation of oleic acid complexes, turbidity of lauric acid complexes, and marked color transformation of fluorescein complexes. As previously shown, DMSO is an aprotic solvent with a relatively reduced dielectric constant (ε), encouraging facilitated ion pairing, and is known to favor ionic interactions between protonatable amines and carboxylic acids [16,18,29]. Therefore, the observed behavior in the dissolution medium DMSO strongly supported successful HIP formation. Furthermore, the purified complexes obtained after lyophilization and washing were further characterized by FT-IR spectroscopy, supporting electrostatic interactions between the primary amino groups of tobramycin and the carboxylic acid groups of the utilized counterions. In Figure 4, pure tobramycin had a broad absorption in the range of 3200–3500 cm^−1^ within the intervals of OH and NH stretching vibrations, which are typical of the aminoglycoside structure. Oleic acid and lauric acid had bright carbonyl (C=O) stretching bands at 1700–1720 cm^−1^ that were associated with free carboxylic acid. The carbonyl region of the spectra of the HIP complexes of tobramycin with oleic acid, lauric acid, and fluorescein also showed significant changes in the spectra compared to the corresponding pure fatty acids. Specifically, attenuation and slight broadening of the carbonyl band were observed, with slight changes observed in the amine-related part of the tobramycin vibrational region. These differences indicate electrostatic interaction between the protonated amine groups of tobramycin and the carboxylate groups of the fatty acid counterions, leading to the formation of non-covalent complexes.

It has been known that hydrophobic ion pairing raises the apparent lipophilicity of hydrophilic ionizable drugs by reversible ionic association with oppositely charged amphiphilic counterions, without seeking the creation of new covalent bonds. Therefore, the evidence of HIP in the FTIR is usually in the form of a peak attenuation, broadening of the bands, or a slight change in the existing functional group regions and not the emergence of new characteristic peaks [12,24,30]. Lack of further peaks that would have shown degradation proves that the chemical structure of tobramycin was not broken after complexation. Comprehensively, the FTIR data allow concluding that hydrophobic ion pairs between tobramycin and fatty acid, along with fluorescein counterions, were successfully formed and did not cause any damage to the structure of the drug, which is critical to retain antibacterial efficacy and the control of physicochemical behavior to employ tobramycin in the oral nano-delivery studies.

### 2.3. In Vitro Drug Release

**Figure 5:**
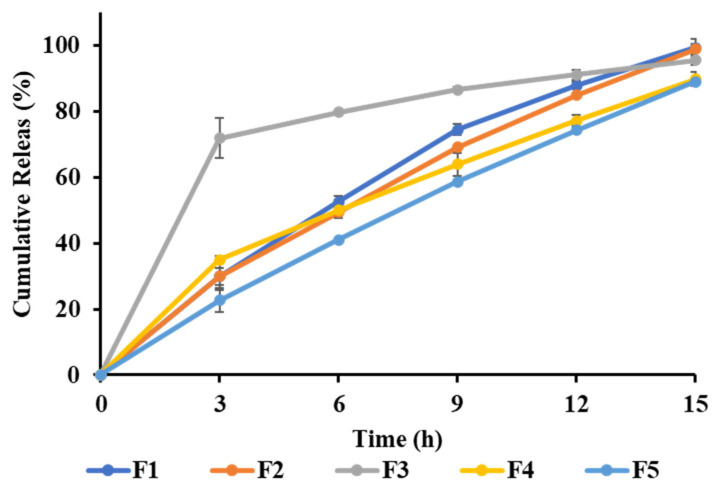
In vitro cumulative release profiles of tobramycin from formulations F1–F5 over 15 h, determined using the dialysis-based release method.

Figure 5 shows the release behavior of the formulations varied clearly, showing that composition did have a pronounced effect on drug diffusion and release kinetics. The formulations of hydrophobic ion-pair (HIP)-based F1 and F2 exhibited more controlled release behavior compared to the non-ion-paired comparator, with cumulative release values of 99.27% and 98.76% at 15 h, respectively. The two formulations exhibited a moderate initial release phase during the first 3 h, followed by a slower, gradual cumulative release across the rest of the points in time. In comparison, F3, the non-ion-paired formulation, exhibited a strong burst release with about 71.79% of the drug being released during the first 3 h. In nano-sized drug delivery systems, burst release is generally associated with rapid diffusion of weakly associated drug molecules near the particle surface, which are localized on the particle surface or are located in the carrier phase as small, weakly associated particles and occur within systems with short diffusion distances and high interfacial area. The controlled-release nano-sized drug delivery systems are hence commonly intended to minimize this initial diffusion-based loss but retain the total drug availability [31]. The reduced initial burst in F1 and F2 indicates that the drugs are more strongly associated when they are hydrophobically bound by ion pairs and have less instant diffusion into the aqueous release medium. Hydrophobic ion pairing is known to alter the apparent lipophilicity and formulation behavior of ionizable hydrophilic drugs by changing the release kinetics using reversible non-covalent association with oppositely charged counter-ions; past reports have demonstrated that HIP-based nanoparticle approaches can be utilized to control the release kinetics and reduce rapid early release and maintain effective overall release of the incorporated drug [30]. This kind of behavior is observed in the current findings, with the HIP-based formulations exhibiting less burst release in comparison with F3 but still obtaining close to complete cumulative release at the culmination of the experiment. Formulations that contained the fluorescein (F4 and F5) exhibited retarded cumulative release (to 89.74% and 88.98% at 15 h, respectively). Since the systems have a different composition compared to the main HIP and non-HIP formulations, the slower release likely reflects formulation-dependent changes in diffusion behavior in the dispersed systems as opposed to ion pairing itself. Oral delivery-wise, reduction in excessive burst release can potentially be beneficial in maintaining the existence of drugs at the gastrointestinal interface and increasing the chance of exposure to the absorptive mucosal surface, which can be beneficial to highly hydrophilic compounds with mucus-related transport barriers [32]. Notably, the cumulative release approaching the entirety of F1 and F2 means that the kinetics of release in a hydrophobic ion pairing depend on an interaction without inhibiting final drug release. Overall, these findings demonstrate that the incorporation of hydrophobic ion pairing effectively reduced the early burst release of tobramycin while maintaining high cumulative drug release over time.

### 2.4. Cytotoxicity Assessment in Caco-2 Cells

**Figure 6:**
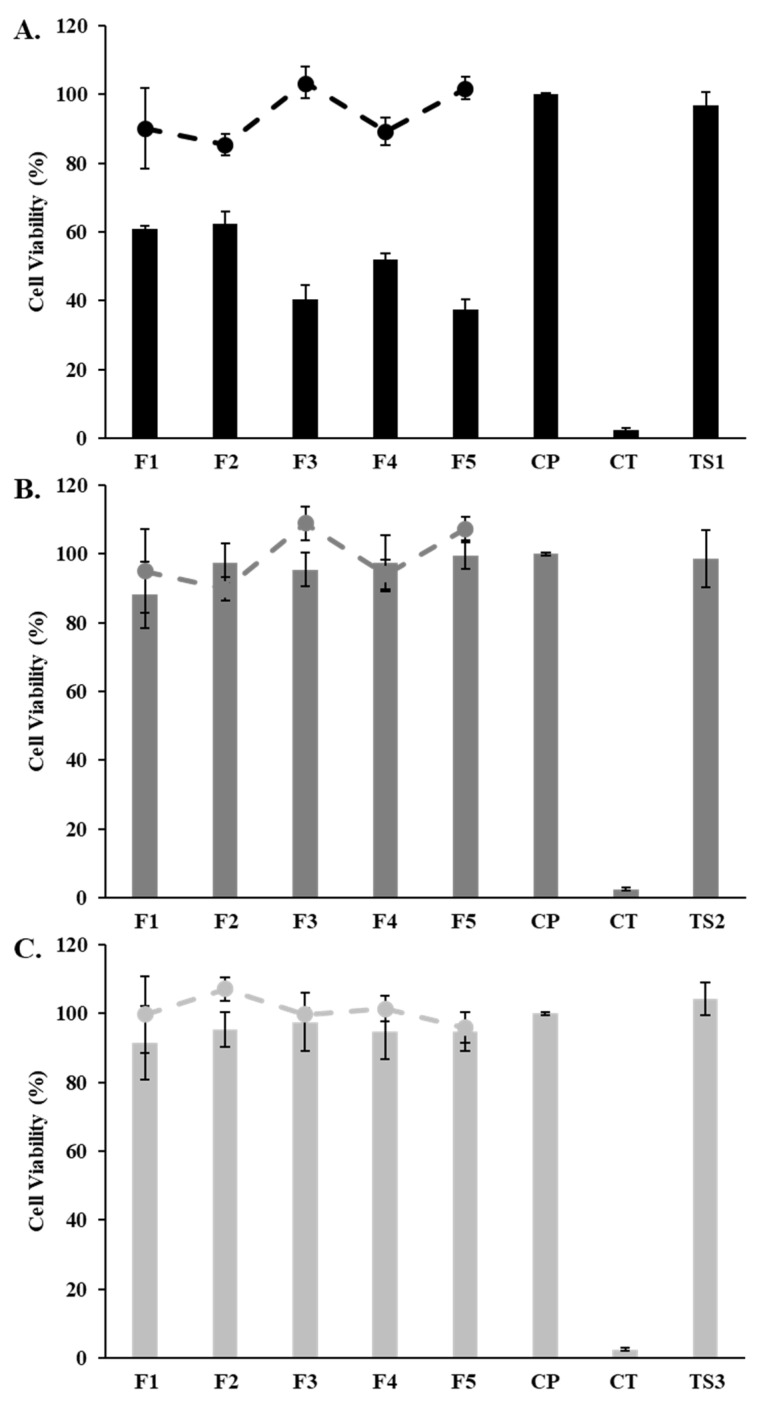
Cell viability of Caco-2 cells after exposure to formulations F1–F5 before (bars) and after (dashed lines) omitting tobramycin at dilution levels of 1:1000 (**A**), 1:10,000 (**B**), and 2:10,000 (**C**), determined using the MTT assay. CP represents the untreated cell control (100% viability), while CT represents the Triton X-100-treated cells. TS1, TS2, and TS3 represent free non-formulated tobramycin solutions used at the same concentrations obtained from F1–F5 dilutions in culture medium at 1:1000, 1:10,000, and 2:10,000, respectively.

The cytotoxicity of the formulations developed was measured in the form of Caco-2 cells through the MTT assay at the end of the exposure duration (approximately 6 h) in the dilutions (1:1000, 1:10,000, and 2:10,000) of the formulations (Figure 6A–C). The cytotoxicity profiles of formulations F1–F5 were evaluated at three dilution levels (1:1000, 1:10,000, and 2:10,000) and compared with their corresponding blank formulations to distinguish the contribution of tobramycin from that of excipients. Across all tested dilutions, the blank formulation components omitting tobramycin consistently maintained high cell viability, typically approaching or exceeding 90%, indicating minimal intrinsic toxicity of the excipient systems under these conditions. In contrast, a marked reduction in cell viability was observed at the 1:1000 dilution for all tobramycin-loaded formulations, with viability values ranging approximately from 37% to 63%, demonstrating pronounced cytotoxicity in the presence of tobramycin.

Statistical analysis revealed that, at the 1:1000 dilution, all tobramycin-loaded formulations exhibited a statistically significant decrease in cell viability compared to their corresponding blank formulations (*p* < 0.05). No significant differences were observed among blank formulations, confirming the negligible cytotoxic contribution of excipients at this dilution [16,17,33,34,35]. Moreover, comparisons across dilution levels demonstrated that cytotoxicity at 1:1000 was significantly greater (*p* < 0.05) than at 1:10,000 and 2:10,000, indicating a clear concentration-dependent effect. At higher dilutions, cell viability was largely preserved (>88–99%), and no statistically significant differences (*p* > 0.05) were observed between loaded and blank formulations, nor among different formulations, suggesting that formulation composition did not significantly influence cytotoxicity under these conditions.

The observed cytotoxicity can be primarily attributed to tobramycin following cellular internalization rather than to the excipients. Aminoglycosides, including tobramycin, are well documented to induce intracellular toxicity upon uptake, particularly in epithelial, neuronal, and renal cells. Mechanistically, tobramycin accumulates intracellularly and has been associated with mitochondrial dysfunction, oxidative stress, and disruption of protein synthesis, ultimately leading to cell damage and death. These effects are well established in nephron cells, where aminoglycosides induce nephrotoxicity through accumulation in proximal tubular cells, and in neuronal systems, where ototoxicity is linked to damage of sensory hair cells in the inner ear [36,37]. Therefore, the concentration-dependent decrease in viability observed in Caco-2 cells strongly supports the hypothesis that the developed formulations enhance cellular uptake of tobramycin, resulting in increased intracellular exposure and consequent cytotoxicity.

Importantly, the contribution of excipients to cytotoxicity at the tested dilutions (1:1000–2:10,000) appears minimal, as evidenced by the high viability observed in all blank formulations. Components such as Tween 80, dimethyl sulfoxide, propylene glycol, fatty acids, and fluorescein are known to exhibit cytotoxic effects only at relatively high concentrations [16,33,34,38,39,40], typically exceeding those present under the studied dilution conditions. Fatty acids such as oleic acid and lauric acid may exert membrane-disruptive effects at elevated concentrations, and fluorescein may exhibit mild phototoxicity under specific conditions [38,39,40]; however, their lack of significant impact in the blank formulations indicates that they do not contribute substantially to cytotoxicity at the concentrations used in this study. Supporting this observation, additional experiments at lower dilutions (1:100 and 1:200) resulted in substantially reduced viability (≤15% and 20–30%, respectively), further confirming a direct relationship between tobramycin concentration and cytotoxicity. Importantly, since the blank formulations did not exhibit comparable toxicity at 1:1000–2:10,000 dilutions, the contribution of excipients to cytotoxicity within this range can be considered negligible. In contrast, at much lower dilutions (e.g., 1:100 and 1:200), where both drug and excipient concentrations are considerably higher, reduced cell viability may arise from combined toxic effects.

The cytotoxicity observed for the tobramycin-loaded formulations is most plausibly attributed to enhanced intracellular delivery mediated by the nano-based drug delivery systems rather than to the intrinsic toxicity of tobramycin in the extracellular environment. Additional proof was obtained from the control experiments using free (non-formulated) tobramycin at equivalent concentrations corresponding to the tested dilutions (1:1000, 1:10,000, and 2:10,000), which maintained high cell viability (96.7 ± 3.9%, 98.6 ± 8.2%, and 104.3 ± 4.8%, respectively), comparable to the 100% positive control. These findings indicate that, in the absence of a delivery vehicle, tobramycin does not exert significant cytotoxic effects under the tested conditions, likely due to its limited passive permeability across cellular membranes [15,41]. In contrast, when incorporated into the NCAs F1–F5, a pronounced and concentration-dependent decrease in cell viability was observed, particularly at the 1:1000 dilution and more prominently at higher concentrations (1:100 and 1:200). This suggests that the formulations facilitate cellular uptake of tobramycin, thereby increasing its intracellular concentration to levels sufficient to induce toxicity. Conversely, at higher dilutions (1:10,000 and 2:10,000), where the effective intracellular drug concentration is presumably lower, cytotoxic effects were absent or markedly reduced. Collectively, these results provide strong evidence that tobramycin-induced cytotoxicity in this system is uptake-dependent and is significantly enhanced by the nano-based delivery vehicles.

Collectively, these findings provide strong evidence that the observed cytotoxicity in this system is predominantly driven by tobramycin in a concentration-dependent and uptake-dependent manner and is primarily enhanced by the nano-based delivery vehicles. This increased intracellular delivery likely underlies the observed toxicity at higher concentrations and supports the utility of these systems as efficient drug delivery vehicles.

### 2.5. Cellular Uptake Studies

**Figure 7:**
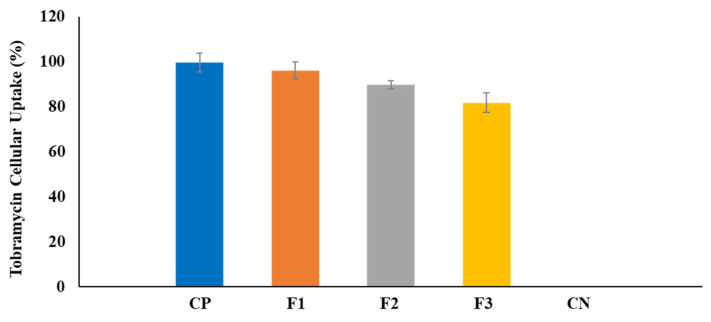
Results of tobramycin cellular uptake following incubation of F1, F2, and F3 2:10,000 dilutions in PBS (pH 7.4) over Caco-2 cells for 5 h at 37 °C. Abbreviations: CP (positive control) and CN (negative control).

Cellular uptake of tobramycin following 5 h incubation with formulations F1–F3 at a dilution of 2:10,000 demonstrated a clear and significant enhancement in intracellular drug levels compared to free tobramycin (Figure 7). The NCAs F1–F3 produced well-defined and quantifiable HPLC peaks at the characteristic retention time (~2.7 min), indicating successful intracellular delivery of tobramycin. In relation to the 100% positive control presenting full cellular uptake, results shown in Figure 7 present tobramycin cellular uptake values of 96.14 ± 3.85%, 89.74 ± 1.89%, and 81.76 ± 4.32% from the formulations F1, F2, and F3, respectively. In contrast, free tobramycin was similar to the negative control (blank buffer-treated cells), and both showed no detectable HPLC peaks at the characteristic retention time of tobramycin (~2.7 min). This proved that free tobramycin in the culture medium exhibited negligible or no cellular uptake in the absence of a delivery system. This difference highlights a statistically significant improvement (p < 0.001) in cellular uptake for all formulations relative to the free drug, supporting the effectiveness of the nano-based delivery vehicles.

The enhanced uptake observed for F1 and F2 can be primarily attributed to ion pairing of tobramycin with fatty acids, which increases its apparent lipophilicity and facilitates interaction with the lipid-rich cellular membrane [18,35]. Specifically, oleic acid (F1) and lauric acid (F2) form hydrophobic ion pairs with the polycationic tobramycin molecule, thereby reducing its polarity and improving membrane permeability [16,42]. In addition, the presence of permeability-enhancing excipients such as Tween 80 and propylene glycol further contributes to increased membrane fluidity and facilitates transcellular transport. In contrast, F3, which contains free tobramycin without ion pairing, exhibited comparatively lower cellular uptake, likely due to its higher hydrophilicity and the relatively rapid drug release profile (≈71.8% within 3 h), which limits sustained interaction with the cell membrane and reduces effective intracellular accumulation [35,43,44].

Physicochemical characteristics of the formulations also played a critical role in enhancing uptake. The nanodroplet sizes for F1 (10.34 ± 0.25 nm), F2 (13.46 ± 0.64 nm), and F3 (11.12 ± 0.75 nm) fall within an optimal range for cellular internalization, allowing efficient interaction with the cell membrane and potential uptake via endocytic pathways [45,46,47,48]. Moreover, the moderately negative zeta potential values (−10.32 mV for F1, −11.13 mV for F2, and −4.99 mV for F3) contribute to colloidal stability while still permitting interaction with the negatively charged cell membrane, particularly when combined with surfactant-mediated membrane perturbation. The slightly higher negative charge of F1 and F2 compared to F3 may also reflect stronger ion pairing and more stable nanostructures, further supporting enhanced uptake [16,49].

The absence of cellular uptake of free tobramycin could be explained by its violation of Lipinski’s Rule of Five [50]. Tobramycin is highly hydrophilic, possesses multiple hydrogen bond donors and acceptors, and carries a strong positive charge at physiological pH, all of which limit passive diffusion across lipid membranes. Its large polar surface area further restricts membrane permeability. In parallel, these uptake findings are consistent with the observed cytotoxicity results above. The utilized free tobramycin solutions in the culture medium at the same concentration from the diluted formulations were non-cytotoxic and produced cell viability profiles comparable to those obtained for the positive control [42,51]. When loaded into the formulation, however, the 1:1000 dilution bearing the same tobramycin concentration induced a significant cytotoxicity. In terms of cellular uptake, this effect was attributed to the increased intracellular accumulation of tobramycin facilitated by the delivery system that was absent in the case of free tobramycin. In contrast, at the current dilution (2:10,000), no significant cytotoxicity was observed despite measurable intracellular uptake, indicating that the delivered drug concentration remained below the toxicity threshold. This confirms that the developed NCAs enhance cellular uptake of tobramycin while allowing modulation of intracellular exposure through dilution, thereby decoupling delivery efficiency from cytotoxic effects. Collectively, these results demonstrate that formulations F1–F3 function as efficient NCAs that significantly improve intracellular delivery of tobramycin compared to the free drug, primarily through modulation of lipophilicity, nanoscale size, and membrane permeability [18,35,42,43]. The current findings demonstrate enhanced cellular uptake in vitro and should be considered preliminary with respect to oral delivery applicability. Further investigations involving transepithelial permeability studies, TEER measurements, and in vivo evaluation are necessary to confirm the potential of these nano-sized systems for improving oral tobramycin delivery.

### 2.6. Fluorescein-Based Uptake Validation: UV–Vis and Confocal Evidence

**Figure 8:**
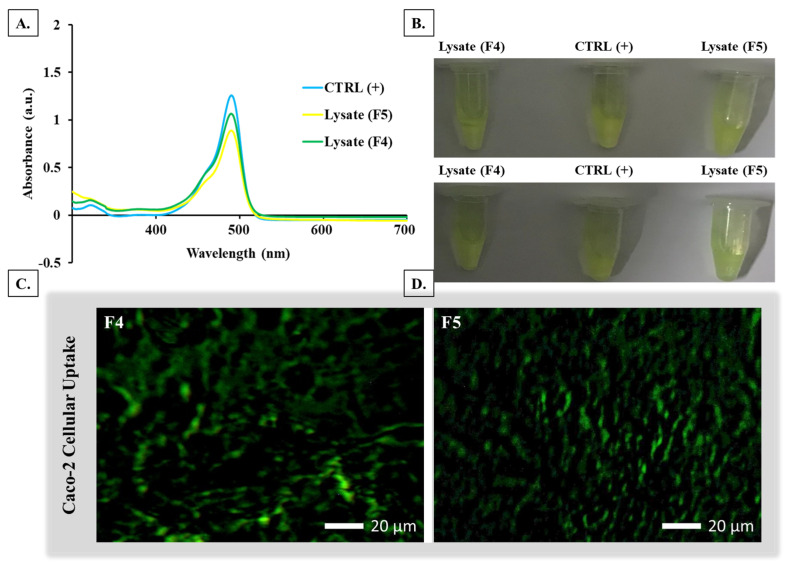
Fluorescein maximum absorbance values at 490 nm obtained from F4 and F5 lysates as compared to the absorbance value of the 100% positive control following cellular uptake by Caco–2 cells (**A**). Pictures showing a physical visualization of fluorescence intensity of the obtained F4 and F5 lysates as compared to the 100% positive control following Caco–2 cellular uptake (**B**) and an instrumental detection of fluorescence intensity (500–550 nm emission and 40× objective lens) following F4 (**C**) and F5 (**D**) incubation for 5 h over Caco–2 cells using confocal microscopic imaging.

Cellular uptake of the fluorescein–tobramycin ion-pair formulations (F4 and F5) was further evaluated using UV–Vis spectroscopy of post-lysis samples and supported by visual fluorescence (Figure 8A–D). Lysates obtained after 5 h incubation at a 2:10,000 dilution exhibited intense green coloration, indicating substantial intracellular delivery of the fluorescein-associated NCAs. UV–Vis scanning (300–700 nm) revealed strong absorbance at ~490 nm, with values of 1.067 for F4 and 0.887 for F5, compared to 1.259 for the corresponding positive control (representing 100% uptake). Based on these values, the relative fluorescein uptake was estimated to be approximately 84.8% for F4 and 70.5% for F5, demonstrating highly efficient intracellular delivery from both formulations.

These findings are consistent with the uptake behavior observed for F1–F3 using HPLC and further confirm the role of the nano-based delivery systems in enhancing tobramycin cellular internalization. Notably, F4 shares the same excipient system as F3 (Tween 80 and DMSO), while F5 aligns with F1/F2 in utilizing Tween 80 and propylene glycol, indicating that both solvent systems effectively support intracellular delivery when combined with appropriate ion pairing. The high uptake efficiency observed at a non-cytotoxic dilution at 2:10,000 further supports that these formulations facilitate substantial intracellular accumulation without compromising cell viability [18,49,52].

Importantly, the UV–Vis results were corroborated by confocal microscopic imaging, which demonstrated intense intracellular fluorescence following treatment with F4 and F5. The observed fluorescence distribution within cells provides qualitative confirmation of uptake and intracellular localization, reinforcing the quantitative UV–Vis findings. Furthermore, in order to eliminate contributions from non-associated extracellular material, all of these quantifications were performed after careful washing of the cells with buffer. Therefore, the quantified signal primarily represented cell-associated uptake mediated by the developed NCAs. Confocal fluorescence imaging and lysate fluorescence recovery further supported substantial intracellular-associated delivery from the fluorescein-containing formulations of similar composition. Similar uptake studies on nano-sized drug delivery systems have quantified cellular uptake based on post-incubation washing followed by cell lysis and analytical quantification of the associated drug fraction, which is commonly interpreted as intracellular-associated nanoparticle uptake [53,54]. Collectively, these results establish that the developed NCAs in this study significantly enhance cellular uptake of both tobramycin and fluorescein, highlighting the effectiveness of the excipient systems (Tween 80, DMSO, and propylene glycol) and ion-pairing strategy in promoting drug internalization.

### 2.7. Antibacterial Activity Assay

**Figure 9:**
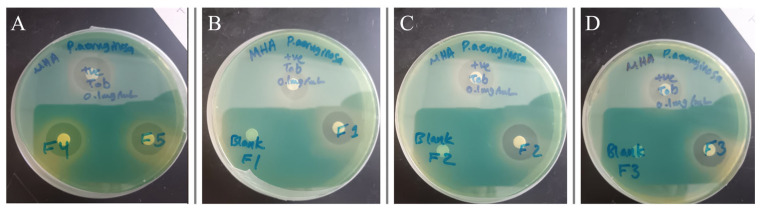
Disc diffusion assay demonstrating the antibacterial activity of tobramycin-loaded formulations F4–F5 (**A**), F1 (**B**), F2 (**C**), and F3 (**D**) against *Pseudomonas aeruginosa* ATCC 9027. The tobramycin solution (free drug control) was included to evaluate the effect of the non-formulated drug, while tobramycin-free formulations (blank formulations) served as negative controls to confirm that excipients did not exhibit intrinsic antibacterial activity. Clear inhibition zones indicate retained antibacterial efficacy after formulation.

The disc diffusion assay (CLSI, 2012) was used to determine the antibacterial activity of the resulting tobramycin formulations against *P. aeruginosa* ATCC 9027 (Figure 9 and Table 1). Clear inhibition zones were observed demarcated around all formulation-loaded discs (F1 to F5). F2 and F3 formulations produced the highest inhibition zones (18 mm) while formulations F1 and F4 produced the lowest (17 mm). The tobramycin solution (Free drug control, 0.214 mM) produced a comparable inhibition zone of 17.5 mm against *P. aeruginosa*. Accordingly, the formulated systems retained the antibacterial activity against this Gram-negative microorganism (Figure 9, Table 1). Conversely, the corresponding tobramycin-free (Blank) formulations did not show any inhibition zones, confirming that the observed activity was attributable to tobramycin rather than the formulation ingredients/excipients [9]. Coupled with the findings, the evidence indicates that the antibacterial activity of tobramycin against *P. aeruginosa* was not observed to be affected by the conduction of the formulation process [55]. This is in line with the established antibacterial spectrum of the aminoglycosides. Tobramycin is considered a potent aminoglycoside with good activity against aerobic Gram-negative bacteria, and historically, it was considered among the preferred aminoglycosides in *Pseudomonas* infections [56]. Its antimicrobial action against *P. aeruginosa* was previously reported and is linked to protein synthesis inhibition caused by tobramycin binding to the 30S ribosomal component of the bacterium [57]. Agar and disk diffusion methods are considered widely accepted comparative tools for in vitro screening of antimicrobial activity, and inhibition zones of tobramycin against *P. aeruginosa* have been reported to be measurable in agar diffusion studies [58].

**Figure 10:**
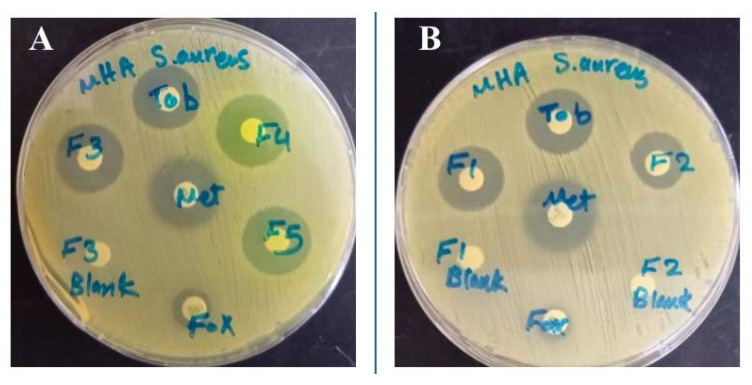
Disc diffusion assay showing the antibacterial activity of the tobramycin-loaded formulations F3–F5 (**A**) and F1–F2 (**B**) against *Staphylococcus aureus*. The tobramycin solution (free drug control) and methicillin comparator discs were used as reference controls, while tobramycin-free formulations (blank formulations) served as negative controls. The presence of measurable inhibition zones confirms preservation of antibacterial activity following formulation.

The disc diffusion assay was also used to determine the antibacterial activity of the formulations developed against *S. aureus* (Figure 10 and Table 1). The inhibition zones were clearly seen around all the formulation discs being tested, whereas the tobramycin-free formulations (Blank formulations) did not exhibit any inhibition zones. This suggests that tobramycin, but not formulation excipients, has the inhibitory effect. The tobramycin solution (free drug control, 0.214 mM) showed an inhibition zone of 17 mm against *S. aureus*, indicating comparable antibacterial activity between the free drug and the tobramycin-loaded formulations, having 17 mm, and F2 having the lowest inhibition zone of 15 mm. The tobramycin solution (free drug control) gave an inhibition zone of about 17 mm, and the control comparator disc of methicillin had an inhibition zone of 16 mm under the studied conditions (Figure 10 and Table 1). In general, the tobramycin-loaded formulations (F1–F5) were inhibitory with similar effects as the tobramycin solution. (free drug control), indicating that the formulation process did not seem to negatively affect the antibacterial effect of tobramycin on *S. aureus*. This observation agrees with the established antibacterial effect of aminoglycosides. Though aminoglycosides are especially known to be active on aerobic Gram-negative bacteria, tobramycin may also exhibit a quantifiable in vitro effect on *S. aureus* [59,60]. The current susceptibility testing work also shows activity of aminoglycosides, including tobramycin, against susceptible *S. aureus* reference strains. Recent studies have shown the antibacterial activity of tobramycin in relation to *S. aureus* reference strains, including ATCC [60,61]. Comprehensively, the findings indicate that all the tobramycin-loaded formulations (F1–F5) maintained antibacterial activity against *S. aureus*, with F4 exhibiting the largest inhibition zone under the experimental conditions.

**Figure 11:**
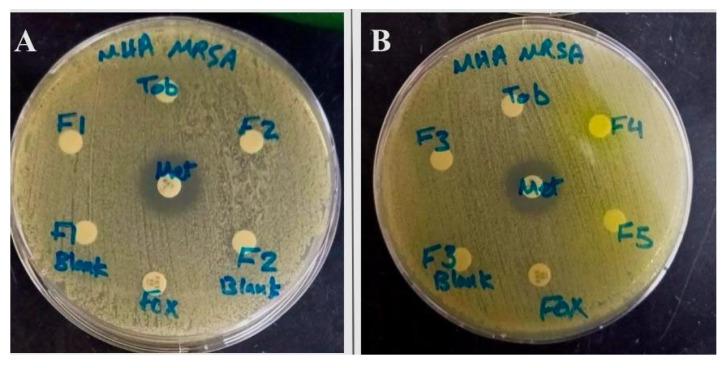
Representative agar disc diffusion assay evaluating the antibacterial activity of the tobramycin-loaded formulations F1–F2 (**A**) and F3–F5 (**B**) against methicillin-resistant *Staphylococcus aureus* (MRSA) ATCC 43300. The tobramycin solution (free drug control) and cefoxitin comparator discs were included as reference controls, while tobramycin-free formulations (blank formulations) served as negative controls. No visible inhibition zones were observed for the formulations under the tested conditions.

The antimicrobial activity of the developed formulations was also evaluated against methicillin-resistant *Staphylococcus aureus* (MRSA) ATCC 43300 using the disc diffusion assay (Figure 11 and Table 1). In the conditions employed in this study, the formulations F1–F5 did not produce visible inhibition zones (NZ) against MRSA. Similarly, the tobramycin-free formulations (Blank formulations) did not produce inhibition zones, confirming that the formulation excipients did not contribute detectable antimicrobial effects under the tested conditions. In the case of MRSA, the developed formulations and the tobramycin control did not produce visible inhibition zones. This finding is broadly consistent with the well-established multidrug-resistant profile of MRSA to various classes of antimicrobials. The clinical challenge of MRSA and the variety of resistance determinants that may restrict the efficacy of traditional antibacterial agents remain among the key points of modern reviews [62]. The lack of inhibition zones indicates that the prepared tobramycin-loaded formulations did not demonstrate detectable anti-MRSA activity under the screening conditions used in this study.

Overall, the formulation components exhibited no intrinsic antimicrobial activity. The tobramycin-loaded formulation had no impact on the antimicrobial spectrum of the drug, as *P. aeruginosa* and *S. aureus* strains that were susceptible to the standard tobramycin solution showed the same response to the formulated tobramycin. But also, the formulation did not enhance the antimicrobial activity of tobramycin, as the formulated drug showed no activity against MRSA and no increase in activity towards *P. aeruginosa* and *S. aureus*, consistent with the standard reference. In addition to the remarkably enhanced tobramycin cellular uptake as previously shown, the formulated NCAs did not compromise their antibacterial activity. These findings support the preservation of the antimicrobial functionality of tobramycin through the use of these NCAs that were developed with the intention of improving its cellular uptake.

## 3. Materials and Methods

### 3.1. Materials

Tobramycin (>98%, Amman Pharmaceutical Industries, Amman, Jordan), oleic acid (99%, extra pure, Alpha Chemika, Mumbai, India), and lauric acid (≥99%, Acros Organics, Geel, Belgium). Polysorbate 80 (Tween 80) and propylene glycol (Sigma-Aldrich, St. Louis, MO, USA). Dimethyl sulfoxide (DMSO: ≥99.9%) and fluorescein (≥95%, ISOLAB, Wertheim, Germany). M Spectrum Laboratories acquired dialysis membranes with a molecular weight cut-off (MWCO) of 12–14 kDa (Rancho Dominguez, CA, USA). Pure water was obtained by using a Milli-Q purification system (Millipore, Burlington, MA, USA). The Caco-2 human colorectal adenocarcinoma cells (ATCC.HTB-37.htb 37) were provided by the University of Jordan (Department of Biopharmacy and Clinical Pharmacy). Dulbecco Modified Eagle Medium (DMEM), fetal bovine serum (FBS), penicillin-streptomycin, and trypsin-EDTA were acquired at Gibco (Thermo Fisher Scientific, Waltham, MA, USA). 3-(4,5-dimethylthiazol-2-yl)-2,5-diphenyltetrazolium bromide (MTT; MW 414.32 g/mol) was bought at Sigma-Aldrich (St. Louis, MO, USA). In the case of antimicrobial testing, Mueller–Hinton agar (MHA) was bought from Oxoid (Basingstoke, UK), and sterile blank filter paper discs (6 mm diameter) were bought from HiMedia Laboratories (Mumbai, India). The tested bacterial strains were *Staphylococcus aureus* ATCC 29213, methicillin-resistant *Staphylococcus aureus* (MRSA) ATCC 43300, and *Pseudomonas aeruginosa* ATCC 9027. The microbiology laboratory at the University of Jordan was used as an institutional repository of these strains. Where necessary, commercial methicillin (5 µg) and cefoxitin (30 µg) discs were used as controls for the antibiotics used. The rest of the reagents and solvents were analytical grade and were used as received.

### 3.2. Preparation of Hydrophobic Ion-Pair (HIP) Complexes

**Figure 12:**
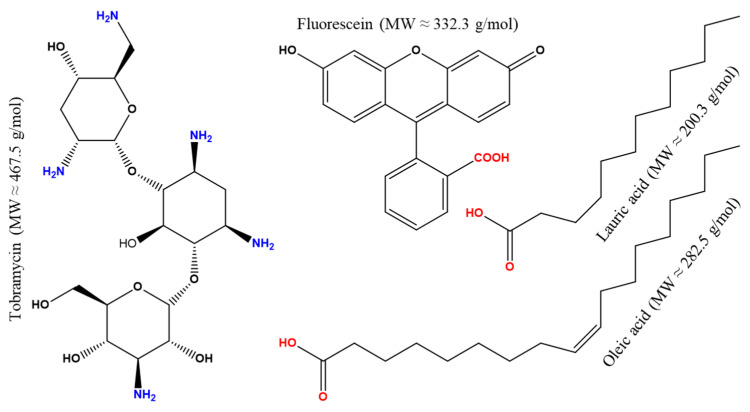
Target HIP complexes with tobramycin.

Tobramycin ion pairs were prepared by complexation with equimolar amounts of oleic acid, lauric acid, and fluorescein, as outlined in formulations F1–F5 (Table 2). Tobramycin (10 mg; MW ≈ 467.5 g/mol) contains five primary amino groups capable of protonation, allowing electrostatic interaction with carboxylate groups from the counterions; therefore, charge-equivalent amounts of oleic acid (30 mg; MW ≈ 282.5 g/mol), lauric acid (20 mg; MW ≈ 200.3 g/mol), and fluorescein (36 mg; MW ≈ 332.3 g/mol) were used (Figure 12) to ensure stoichiometric balance and efficient ion pair formation. Complexation was performed in dimethyl sulfoxide (DMSO), where each component was initially dissolved separately, followed by dropwise addition of the counterion solution to the tobramycin solution under continuous shaking at room temperature. The process resulted in distinct physicochemical changes depending on the counterion, including immediate precipitation with oleic acid, formation of a cloudy dispersion with lauric acid, and a color transition from greenish to red with fluorescein. The resulting ion pair solutions were subsequently frozen at −80 °C and lyophilized under reduced pressure (<10 mbar) to remove DMSO, yielding solid complexes that were used for subsequent formulation development [12,63].

### 3.3. Preparation of Tobramycin HIP-Based NCA Formulations

The dried tobramycin ion pairs obtained as described above were incorporated into NCA preconcentrates by mixing with the respective excipients according to the compositions (*w*/*w* %) outlined for formulations F1–F5 (Table 2), yielding a final mass of 700 mg per formulation in 2 mL Eppendorf tubes. Initial dissolution of the ion pairs was achieved using the cosolvent system of each formulation (either DMSO or propylene glycol) under modest heating, followed by the addition of the remaining excipients with continued mixing under mild heating conditions. In formulations F1, F2, and F5, where incomplete dissolution of the ion pairs was observed, 500 µL of methanol was added to facilitate complete solubilization, and the mixtures were further homogenized. The tubes were subsequently incubated under thermomixing at 37 °C overnight with open lids to allow evaporation of methanol. Following solvent removal, all formulations were obtained as homogeneous, clear preconcentrates containing fully dissolved ion pairs.

### 3.4. Particle Size, Polydispersity Index, and Zeta Potential

The particle size, polydispersity index (PDI), and zeta potential of the developed formulations were determined using DLS with a Zetasizer Nano series instrument (Malvern Instruments Ltd., Worcestershire, UK). The preconcentrates of formulations F1–F5 were diluted 1:100 in aqueous medium at pH 7.4 on an incubated shaker operating at 37 °C prior to analysis to allow spontaneous formation of nanodroplets. Particle size and PDI measurements were performed at 0 h and 24 h to assess temporal stability. Zeta potential measurements were conducted using disposable folded capillary cells, while particle size and PDI were measured using disposable polystyrene cuvettes.

### 3.5. HPLC Analysis

#### 3.5.1. Method of Tobramycin HPLC Analysis

Quantitative analysis of tobramycin was performed using a Shimadzu HPLC system (Shimadzu 7000 series, Kyoto, Japan) equipped with a UV detector. Chromatographic separation was achieved using a reversed-phase C18 column (LiChrospher^®^ 100 RP-18, LiChroCART^®^ 125-4, 125 mm × 4 mm, 5 µm particle size, 100 Å pore size; Merck KGaA, Darmstadt, Germany), consisting of a spherical silica-based stationary phase functionalized with octadecylsilane groups.

The mobile phase was prepared as a mixture of methanol, Milli-Q water, and acetic acid in a volumetric ratio of 20:75:5 (*v*/*v*/*v*), obtained by combining 200 mL of methanol, 750 mL of Milli-Q water, and 50 mL of acetic acid to a final volume of 1 L. The mobile phase with the indicated solvent ratios was filtered, ultrasonicated, and degassed. The system was operated under isocratic conditions with a flow rate of 1.0 mL/min and an average operating pressure of approximately 133 bar. The total run time was 10 min per injection. Detection was carried out at 267 nm with an injection volume of 10 µL. According to this method, despite the relatively weak intrinsic UV absorbance of tobramycin, direct UV detection at 267 nm under highly acidic mobile phase conditions enhanced an efficient protonation of tobramycin and provided reproducible and concentration-dependent chromatographic responses without derivatization. Under these conditions, tobramycin exhibited a retention time of approximately 2.7 min (range: 2.59–2.77 min), with consistent peak symmetry across all tested concentrations.

#### 3.5.2. Calibration Curve and Linearity

**Figure 13:**
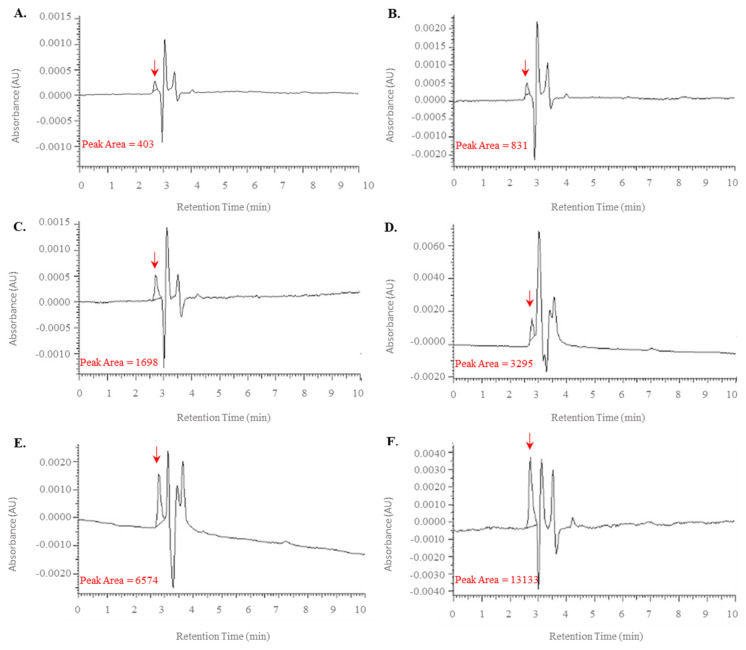
HPLC chromatograms showing tobramycin concentration-dependent peaks ((**A**–**F**): 4.375, 8.75, 17.5, 35, 70, and 140 µg/mL) depicted approximately at 2.7 min retention time using a mobile phase of methanol, Milli–Q water, and acetic acid in a volumetric ratio of 20:75:5 (*v*/*v*/*v*). Red arrows are explained within the figure as “Peak Area” highlighted in red as well.

**Figure 14:**
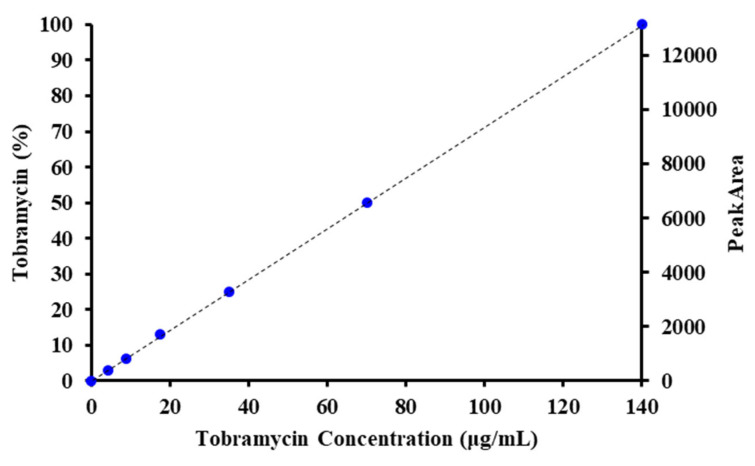
Tobramycin calibration curve illustrated as tobramycin percentage (left Y-axis) and HPLC peak area (right Y-axis) against tobramycin concentration in μg/mL.

**Figure 15:**
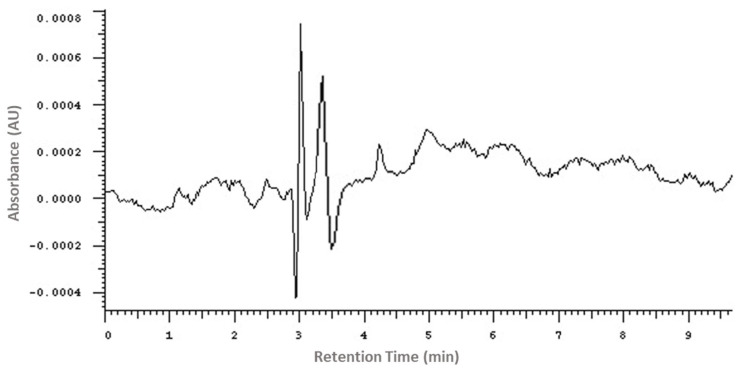
The HPLC chromatogram obtained for the used blank mobile phase was composed of 20:75:5 (*v*/*v*/*v*) methanol, Milli–Q water, and acetic acid.

The concentration-dependent response of tobramycin at a retention time of approximately 2.7 min was established through calibration (Figure 13). Calibration standards were prepared by dissolving tobramycin in the mobile phase to ensure matrix matching. Standard solutions were prepared at concentrations of 4.375, 8.75, 17.5, 35, 70, and 140 µg/mL, yielding peak areas of 403, 831, 1698, 3295, 6574, and 13,133, respectively. A linear relationship between peak area and concentration was observed over the tested range (Figure 14), and linear regression analysis produced the calibration equation Peak Area = 94.1 × C − 15.0, where C is the concentration in µg/mL. The method demonstrated excellent linearity with a correlation coefficient (R^2^) of approximately 0.999. A blank mobile phase injection showed no detectable peak at the retention time of tobramycin, confirming the selectivity of the method. However, sharp and consistent peaks at approximately ≥3 min (Figure 15) of the blank mobile phase (comprising 5% acetic acid) remained unchanged across all runs. The HPLC method also used blank formulation matrices containing excipients, including DMSO, Tween 80, propylene glycol, and fatty acid components, to check its selectivity. Similarly, no interfering peaks were detected at the retention time of tobramycin (~2.7 min). Therefore, the constant sharp peak observed at ≥3 min presented by the blank mobile phase injections was attributed to the acetic acid-containing mobile phase. Moreover, the tobramycin peak maintained consistent retention time, peak shape, and concentration-dependent peak area responses throughout calibration and uptake studies, supporting the selectivity and reliability of the method for tobramycin quantification.

#### 3.5.3. Sensitivity and Method Performance

The sensitivity of the method was evaluated in terms of the limit of detection (LOD) and limit of quantification (LOQ) according to ICH guidelines, based on the standard deviation of the response and the slope of the calibration curve. Based on experimental observations, the LOD was determined to be in the range of approximately 0.177–0.355 µg/mL, corresponding to detectable peaks with surface areas between 22 and 57 units at the characteristic retention time (~2.7 min). The LOQ was estimated to be in the range of approximately 0.53–1.07 µg/mL, calculated as 3.3-fold higher than the LOD. The method demonstrated reliable detection of tobramycin at low concentrations, with peak area increasing proportionally with concentration and a consistent peak shape observed across the tested range. The observed sensitivity and reproducibility are attributed, in part, to the acidic mobile phase conditions, where acetic acid promotes protonation of tobramycin, enhancing its chromatographic response and contributing to stable and well-defined peaks. The method exhibited robust analytical performance, with a stable retention time centered around 2.7 min and strong concentration-dependent peak response.

### 3.6. In Vitro Drug Release Study

In vitro diffusion of tobramycin through the prepared formulations was measured through a dialysis bag diffusion method. In our study, the drug release method used was conducted using a phosphate buffer at pH 7.4, which simulates the near-neutral intestinal physiological pH and ionic aqueous environment despite lacking additional intestinal components such as digestive enzymes, bile salts, and mucus. The ultra-pure water was used to wet the dialysis membranes (molecular weight cut-off 12–14 kDa) overnight. Pre-hydrated dialysis bags were filled with aliquots obtained from previously prepared dilutions for the formulations F1–F5 in phosphate buffer (pH 7.4), and the bags were sealed. Each dialysis bag was placed in a vial filled with 20 mL release medium (phosphate buffer, pH 7.4), and a final dilution of 1:100 was obtained. The vials afterward were kept incubated at 37 ± 0.5 °C in a plate shaker (Shanghai Zhichu Instrument Co., Ltd., Shanghai, China) at 50 rpm. At predetermined time points (0, 0.5, 1, 3, 6, 9, 12, and 15 h), aliquots of 100 µL were taken out of the release medium and put into HPLC vials. Equal volumes of fresh prewarmed release medium were added to it. Vials containing the aliquoted samples were mixed with methanol (200 µL), water (650 µL), and acetic acid (50 µL) to ensure matching the ratios used in the mobile phase. The concentration of tobramycin in every sample was quantified by the validated HPLC technique in Section 2.5. The percentage of drug release was computed cumulatively using Equation (1):

(1) $$Cumulative\;drug\;release\;\left(\%\right)=\frac{Mt}{M_{o}}\times 100$$

in which *Mt* is the amount of Tobramycin released at time *t* and *M_o_* is the initial amount of Tobramycin in the formulation.

### 3.7. Fourier Transform Infrared (FTIR) Test

The ion pairs obtained above as precipitates in 2 mL Eppendorf tubes were centrifuged in acetone, ethyl acetate, and water to remove excess free fatty acids, fluorescein, and tobramycin. An FTIR spectrophotometer (Bruker Corporation, Billerica, MA, USA) was used to record the pure tobramycin, oleic acid, lauric acid, fluorescein, and related ion pairs. In the preparation of samples, the potassium bromide (KBr) pellet method was used. In short, the samples were finely triturated with spectroscopic-grade KBr and pressed under hydraulic pressure into a transparent pellet. The spectra were measured at a wavelength range of 4000–400 cm^−1^ with >20 scans and a resolution of 4 cm^−1^.

### 3.8. In Vitro Cytotoxicity Assessment by MTT Assay

Mitochondrial toxicity of the formulations that were developed was assessed in Caco-2 cells by the use of the MTT assay in a 96-well plate format. The test was conducted within the University of Jordan and a microtiter-plate-based cell viability workflow in line with the previous methodologies reported in the same university and other similar studies [64,65]. Caco-2 cells were planted in 96-well plates and left to attach according to normal cell culture procedures. The studied formulations F1–F5 were diluted in a culture medium of phosphate-buffered saline (PBS) at pH 7.4 to 1:1000, 2:10,000, and 1:10,000, and 250 uL of each diluted formulation was put in the wells. The dilutions were then incubated on cells over a period of about 6 h at 37 °C. The viability of the cells was determined using the MTT assay following the exposure period. Untreated cells were used as the positive control (CP), representing 100% viability, while wells containing a control of Triton X 100 (CT) in the culture medium represented complete cell death. The test was also controlled using tobramycin solutions (TS) as a naked drug prepared at the same concentrations utilized in the above dilutions from the formulations F1–F5. Under the same experimental conditions, blank formulations containing all formulation excipients and counterions but omitting tobramycin were similarly evaluated over Caco-2 cells. The absorbance of the samples was measured at 570 nm using a microplate reader, and cell viability was calculated according to Equation (2):

(2) $$Viable\;Cells\left(\%\right)=\frac{Average\;absorbance\;of\;treated\;wells}{Average\;absorbance\;of\;positive\;control}\;\times \;100$$

### 3.9. Cellular Uptake Studies in Caco-2 Cells

Cellular uptake of tobramycin from formulations F1–F5 was evaluated using Caco-2 cells under standard culture conditions (37 °C, 5% CO_2_, humidified atmosphere). Cells were seeded in appropriate multiwell plates (24-well) and allowed to reach full confluence and differentiation prior to experimentation, with culture medium replaced every 2–3 days. Prior to the uptake study, cells were gently washed with pre-warmed phosphate-buffered saline (PBS, pH 7.4). The formulations were diluted 2:10,000 in serum-free culture medium (pH 7.4) and pre-equilibrated at 37 °C to ensure nanodroplet formation. The diluted formulations were then applied to the cells and incubated for 5 h under standard conditions.

Following incubation, the cells were washed thoroughly with cold PBS to remove extracellular and loosely bound material, and cell lysis was performed using an appropriate lysis buffer (0.5% Triton X-100 in PBS). The resulting lysates were collected and analyzed for tobramycin content using the validated HPLC method described above. Quantification was performed for formulations F1–F3, representing tobramycin ion pairs with oleic acid and lauric acid, as well as free tobramycin. A positive control was prepared by dissolving the total amount of tobramycin corresponding to each formulation dilution in the methanol-containing mobile phase, representing 100% recovery, while blank culture medium served as the negative control.

Formulations F4 and F5, containing the tobramycin–fluorescein ion pair, were utilized to facilitate visualization and complementary quantification of cellular uptake of the nanodroplet delivery system. These formulations enabled subsequent confocal microscopy imaging to assess intracellular localization, as well as spectroscopic determination of fluorescein absorbance in cell lysates following uptake and lysis with Triton X-100, as described in the following sections.

### 3.10. Confocal Microscopic Imaging of Cellular Uptake

Cellular uptake and intracellular localization of the fluorescein-labeled formulations (F4 and F5) were visualized using confocal laser scanning microscopy. Caco-2 cells were seeded in 24-well plates on sterile glass coverslips and cultured under standard conditions (37 °C, 5% CO_2_) until confluence. The cells were then incubated with diluted formulations (2:10,000 in culture medium, pH 7.4) for 5 h at 37 °C, consistent with the uptake protocol. Following incubation, the cells were washed thoroughly with phosphate-buffered saline to remove excess extracellular formulation and subsequently fixed with 4% paraformaldehyde. The coverslips were mounted onto glass slides for imaging.

Fluorescence imaging was performed using a confocal laser scanning microscope (Leica Microsystems, TCS SP8, Wetzlar, Germany). Excitation was set at 488 nm, and emission was collected in the range of 500–550 nm, corresponding to the fluorescence characteristics of fluorescein. Images were acquired using a 40× objective lens, allowing clear visualization of intracellular fluorescence distribution. These conditions enabled qualitative assessment of cellular internalization and localization of the fluorescein-associated nanodroplet delivery system.

### 3.11. UV–Vis Analysis of Fluorescein in Cell Lysates

Following cellular uptake experiments, selected lysates obtained from wells treated with formulations F4 and F5 were collected into 1.5 mL Eppendorf microtubes for spectroscopic analysis. The absorbance of fluorescein associated with the internalized drug delivery system was measured at 490 nm using a UV–Vis spectrophotometer (Shimadzu UV-1900, Kyoto, Japan). In addition, full spectral scans were recorded over the wavelength range of 300–700 nm to characterize the absorption profile of fluorescein in the lysates and to confirm signal specificity.

For qualitative assessment, representative images of selected lysate samples were acquired to visually document the fluorescence intensity resulting from cellular uptake of the tobramycin–fluorescein ion pair in formulations F4 and F5. These samples were compared against corresponding positive controls prepared at the same dilution (2:10,000), representing theoretical maximum uptake. The positive control solutions were obtained by dissolving the respective preconcentrates initially in 200 µL methanol, followed by dilution to a final volume of 10 mL with buffer at pH 7.4. Both absorbance measurements and visual fluorescence intensity of lysates were evaluated relative to these controls to provide complementary quantitative and qualitative evidence of cellular uptake.

### 3.12. Antimicrobial Activity by Agar Disc Diffusion Assay

The agar disc diffusion assay was used in testing the antimicrobial activity of the formulations developed on Mueller–Hinton agar (MHA) plates. The study was carried out to enable a comparative analysis of the inhibitory effect of the formulations of tobramycin prepared on the distinct bacterial and fungal strains. Gram-positive bacteria chosen were Staphylococcus aureus ATCC 29213 (*S. aureus*) and methicillin-resistant Staphylococcus aureus (MRSA) ATCC 43300, and Gram-negative bacteria chosen were Pseudomonas aeruginosa ATCC 9027 (*P. aeruginosa*). They are reference strains that frequently appear in the antimicrobial susceptibility studies and were provided by institutions in the microbiology lab at the University of Jordan. General principles of antimicrobial disc diffusion assays developed in the literature were utilized in carrying out the experimental procedure [66,67,68]. Even microbial lawns were formulated and inoculated on the Mueller–Hinton agar plates with sterile cotton swabs onto fresh overnight microbial cultures. The tested formulations were carried as sterile blank paper discs (6 mm diameter). The antimicrobial assay was carried out using fresh 1:100 dilutions (~0.214 mM tobramycin) of the studied formulations F1–F5. To every preparation, 20 µL of the prepared sample was placed on a sterile blank disc and left to dry before laying the disc on the inoculated agar surface. Negative controls were blank formulation discs to determine possible antimicrobial effects of formulation excipients. To validate the presence of the antibacterial activity, there was a positive control of tobramycin. In addition, commercial antibiotic discs such as methicillin (5 µg) and cefoxitin (30 µg) were provided as comparator controls of Staphylococcus species and MRSA, where necessary. The plates inoculated were incubated overnight in aerobic conditions, and the size of the diameter of the inhibition zones around each disc in millimeters (mm) was obtained after the conclusion of this period. The overall inhibition zone, which also involved disc diameter, was noted. When no zone was observed on the halo of the inhibition, the outcome was noted as no zone (NZ). The disc diffusion assay was performed to offer a comparative screening analysis of the antimicrobial activity within the tested formulations and not to ascertain clinical susceptibility breakpoints. Once and under the mentioned conditions, experiments to determine the qualitative antibacterial activity of the formulations were performed.

### 3.13. Statistical Analysis

All experimental data were expressed as mean ± SD (n ≥ 3). The HPLC calibration curve showed adequate linearity, with a correlation coefficient (R^2^) of approximately ~0.999. Statistical comparisons between groups were performed using one-way analysis of variance (ANOVA) followed by Tukey’s post hoc test to evaluate multiple group differences, with a significance level set at *p* < 0.05. In cases of pronounced differences, highly significant values (*p* < 0.001) were observed. The data processing and statistical testing followed Microsoft Excel and GraphPad Prism version 10, where necessary.

## 4. Conclusions

In this study, NCAs incorporating tobramycin ion pairs were successfully developed and systematically evaluated. The formulations (F1–F5) exhibited nanoscale droplet sizes (9.50 to 16.30 nm), colloidal stability with narrow distributions (PDI < 0.3), and moderate negative zeta potentials (−4.99 to −11.13 mV), indicating efficient ionic interaction with tobramycin primary amino groups and suitability for cellular interaction. Drug release studies demonstrated formulation-dependent behavior, with sustained release observed for ion-paired systems, while free tobramycin formulations showed faster release profiles. Importantly, all formulations exhibited high biocompatibility at appropriate dilutions (1:10,000 and 2:10,000), maintaining near-complete cell viability, while concentration-dependent cytotoxicity at higher doses confirmed effective intracellular delivery. Cellular uptake studies revealed a significant enhancement in tobramycin internalization when the studied NCAs (*p* < 0.001) were compared to the free drug, which showed negligible or undetectable uptake. This was quantitatively confirmed by HPLC analysis for F1–F3 and further supported by fluorescein-based tracking in F4 and F5. The latter demonstrated strong intracellular fluorescence, visually evident in lysates; quantitatively supported by high UV-Vis absorbance values (70.5–84.8% relative to positive control); and qualitatively confirmed by confocal microscopy imaging. The substantial uptake of fluorescein from F4 and F5 provides additional indirect evidence supporting efficient intracellular delivery of tobramycin in analogous ion-paired systems with oleic and lauric acids. The antimicrobial evaluation further confirmed that all tobramycin-loaded formulations (F1–F5) retained potent antibacterial activity, exhibiting inhibition zones comparable to free tobramycin against *P. aeruginosa* and *S. aureus*. In contrast, the corresponding blank formulations showed no detectable activity, confirming that the antimicrobial effect is solely attributed to tobramycin. These findings demonstrate that ion pairing and NCA formulations did not compromise the intrinsic antibacterial efficacy of tobramycin while enabling enhanced delivery properties. Overall, the developed NCAs effectively enhanced the cellular uptake of tobramycin through ion pairing and the use of permeability-enhancing excipients, demonstrating both functional efficiency and formulation elegance. These findings highlight the potential of this delivery strategy to overcome biological barriers associated with tobramycin absorption while maintaining its antimicrobial activities to the optimum level. Consequently, this platform offers promising prospects for future in vivo studies aimed at improving oral bioavailability and enabling the transition of tobramycin therapy from parenteral to oral administration.

## Figures and Tables

**Table 1:** Inhibition zone diameters (mm) of the tested formulations and control discs against the studied bacterial strains using the agar disc diffusion assay.

Sample	*P. aeruginosa* ATCC 9027	*S. aureus* ATCC 29213	MRSA ATCC 43300
**F1**	17	17	NZ
**F1 blank**	NZ	NZ	NZ
**F2**	18	15	NZ
**F2 blank**	NZ	NZ	NZ
**F3**	18	17	NZ
**F3 blank**	NZ	NZ	NZ
**F4**	16	18	NZ
**F4 blank**	—	—	—
**F5**	16	17	NZ
**F5 blank**	—	—	—
**Tobramycin (0.214 mM)**	17.5	17	NZ
**Tobramycin (2.14 mM)**	—	—	NZ
**Methicillin (5 µg)**	—	16	15
**Cefoxitin (30 µg)**	—	7	NZ

**NZ**: No visible inhibition Zone. —: not tested/not recorded.

**Table 2:** Composition of tobramycin NCA formulations (F1–F5).

Formulation	Tobramycin (mg)	Fatty Acid (mg)	Tween^®^80 (mg)	Propylene Glycol (mg)	DMSO (mg)	Fluorescein (mg)
**F1**	10	Oleic acid (30)	200	460	—	—
**F2**	10	Lauric acid (20)	200	470	—	—
**F3**	10	—	140	—	550	—
**F4**	10	—	140	—	514	36
**F5**	10	—	140	514	—	36

## Data Availability

The data supporting the findings of the article are available within this article. All figures, including the graphical abstract, are original to this article and were neither imported nor reproduced from any other source.
